# A biologically inspired neural network controller for ballistic arm movements

**DOI:** 10.1186/1743-0003-4-33

**Published:** 2007-09-03

**Authors:** Ivan Bernabucci, Silvia Conforto, Marco Capozza, Neri Accornero, Maurizio Schmid, Tommaso D'Alessio

**Affiliations:** 1 Dipartimento di Elettronica Applicata, Università degli Studi "Roma TRE", Roma, Italy; 2 Dipartimento di Scienze Neurologiche, Università "La Sapienza", Roma, Italy

## Abstract

**Background:**

In humans, the implementation of multijoint tasks of the arm implies a highly complex integration of sensory information, sensorimotor transformations and motor planning. Computational models can be profitably used to better understand the mechanisms sub-serving motor control, thus providing useful perspectives and investigating different control hypotheses. To this purpose, the use of Artificial Neural Networks has been proposed to represent and interpret the movement of upper limb. In this paper, a neural network approach to the modelling of the motor control of a human arm during planar ballistic movements is presented.

**Methods:**

The developed system is composed of three main computational blocks: 1) a parallel distributed learning scheme that aims at simulating the internal inverse model in the trajectory formation process; 2) a pulse generator, which is responsible for the creation of muscular synergies; and 3) a limb model based on two joints (two degrees of freedom) and six muscle-like actuators, that can accommodate for the biomechanical parameters of the arm. The learning paradigm of the neural controller is based on a pure exploration of the working space with no feedback signal. Kinematics provided by the system have been compared with those obtained in literature from experimental data of humans.

**Results:**

The model reproduces kinematics of arm movements, with bell-shaped wrist velocity profiles and approximately straight trajectories, and gives rise to the generation of synergies for the execution of movements. The model allows achieving amplitude and direction errors of respectively 0.52 cm and 0.2 radians.

Curvature values are similar to those encountered in experimental measures with humans.

The neural controller also manages environmental modifications such as the insertion of different force fields acting on the end-effector.

**Conclusion:**

The proposed system has been shown to properly simulate the development of internal models and to control the generation and execution of ballistic planar arm movements. Since the neural controller learns to manage movements on the basis of kinematic information and arm characteristics, it could in perspective command a neuroprosthesis instead of a biomechanical model of a human upper limb, and it could thus give rise to novel rehabilitation techniques.

## Background

Human beings are able to accomplish extremely complex motor tasks in all kinds of environments by means of a highly organized architecture including sensors, processing units and actuators. From a cognitive and developmental perspective, and a rehabilitation standpoint, it is necessary to fully understand the complex interactions between the controller (the Central Nervous System) and the controlled object (all parts of the body)[1]. These interactions describe the process of motor control for which many theories have been developed. As far as the generation of motor commands is concerned, in literature it is generally acknowledged that nervous system generates motor commands based on internal models able to take account of the kinematics and the dynamics of the biomechanical structures [2-4]. These models can be described as groups of neural connections that intrinsically contain information about biomechanical properties of the human body in relation both to the environment and the subject's experience.

However, the mechanisms underlying the generation and organization of these neural models are still object of controversy [5]. In order to interpret their functions, in literature different computational approaches to simulate both the biomechanical structure and the controller have been presented for a 2D [6] framework.

In this context, there is an interest in the use of Artificial Neural Networks (ANN) because of their capabilities to adapt and to generalise to new situations. In order to link the neural learning/adaptation processes to their artificial replica, ANN have been used in some studies regarding neurophysiologic simulations. However, most of these ANN imply the presence of a supervisor that uses sensory information in order to minimize the error related to the motor task [7]. This methodology, commonly implemented on forward multilayer networks with retrospective learning (back propagation), is efficient from an operative standpoint, but not completely plausible as a biologically inspired learning model of motor control, at least for the presence of a teacher who is pre-existent to the organization of the system.

To overcome this drawback, neural models using unsupervised training techniques for the exploration of motor spaces have been proposed [8] thus meeting the features of self-organization typical of internal representations. The adaptability of the neural model together with the unsupervised training can also answer to environmental modifications such as those represented by external force fields and haptic distortions. Following this approach, it is of interest to study models able to simulate motor control mechanisms in terms of both generating and managing the sequence of motor commands that enable the arm to execute movements in the space. In this paper the focus is on the execution of ballistic movements.

According to the work of Karniel and Inbar [9], ballistic movements can be studied considering that: 1) there is no visual information; 2) any single movement is ballistic. As for every voluntary movement, the central nervous system must address three main computational problems: 1) determination of the desired trajectory in the visual coordinates; 2) transformation of the trajectory from visual to body coordinates; 3) generation of motor commands [10]. The lack of visual information and the ballistic nature prevent to have a feedback on the controller [11,12]: in fact, the delay introduced by a proprioceptive feedback in a biological system is too large to permit on-line corrections of the trajectories, and other studies [13] state that motor commands could be adjusted online without the need to involve a conscious decision process.

In any case, the commonly accepted idea is that ballistic movements can be managed by feed-forward controllers without using visual information as feedback. Some common characteristics are generally shared by ballistic movements on a plane, and these are: roughly straight pathways and bell-shaped hand speed profiles [14,15]. Moreover, point to point movements have been studied following the hypothesis known as the minimum variance rule, able to attain physiological kinematic results as Fitt's Law and 2/3 Power Law [16]. Some authors [17,18] tried to provide a mathematical explanation of these kinematic invariants suggesting the hypothesis that the central nervous system aims at maximizing the smoothness of the movement.

In this work, ballistic movements will be controlled by an ANN controller that can be defined as "biologically inspired". It will be able to generate muscular activations knowing only the starting and arrival points of each movement, giving rise to a solution for the inverse dynamics problem (that is determining muscular forces on the basis of kinematic information). The muscular activations will generate ballistic movements having characteristics similar to human movements. This biologically inspired model will integrate an ANN, which should accomplish the task on the basis of its adaptability and plasticity [19,20], together with a biomechanical arm model, considered as a 2 DOF system, in order to simulate the behaviour of an end-effector driven by the sequences generated by the controller.

In the first part of this work, materials and methods will be reported: after a description of the parallel distributed computational system that has been used, the generator of the neural input commands and the biomechanical model of the arm will be presented. Finally, the evaluation tests and the obtained results will be discussed.

## Methods

In this section we describe the general scheme of the proposed model, which can be divided into three main modules, each one with a specific functionality in the transformation process from perception to motor action, that is: the perception task, the elaboration of data and the motor activation. Therefore, two computational blocks simulate the motor control of the upper limb, while a third block is responsible for the modelling of the actuator.

The first module is devoted to processing spatial information in order to solve the inverse dynamics problem (i.e. which neural signals, that is which forces, have to be generated to reach a specific point in the environment?). The strategy can be acquired after a series of synaptic modifications that represent the construction of the internal model both in architectural and functional ways. The whole process, that simulates the generation of the internal models by means of synaptic modifications, is called learning. It must be emphasized that, since the main purpose of the present work is to characterize a model simulating the generation and the actuation of ballistic movements, no online feedback on the position error is present in the scheme. We deal, in fact, with a process where the learning scheme modifies the neural features in order to map the working space and reach the desired targets. Even if the learning scheme can be considered as a functionality of the Neural System, a separate paragraph in the Materials and Methods section has been devoted to the explanation of the learning process in order to outline the processing scheme adopted.

The second module is called Pulse Generator, and it essentially generates the motor signals necessary for to activate the muscles and to consequently produce the movements of the arm model.

The third module simulates a simplified version of the biomechanical arm model. In fact, the human arm presents a high number of degrees of freedom and a redundancy due to the difference of dimensions between muscular activations space and working space (that is the whole set of the points attainable by the arm model), so that the set of available ways to accomplish a specific task is not unique. In the model, only two mono-articular pairs of muscles for each joint (elbow and shoulder) and a bi-articular pair of muscles connecting the two joints have been taken into account. The first agonist-antagonist pair acts across the shoulder joint: the pectoralis major is the flexor, while the deltoid is the extensor. The second pair acts across the elbow joint: the long head biceps brachialis is the flexor, while the lateral head triceps brachialis is the extensor. The third pair of muscles links both the joints: the flexor is the biceps brachialis short head and the extensor is the triceps brachialis long head.

From the results that will be presented below, it emerges that, even in this simplified version, the synthesized system is able to execute accurate planar movements.

### The proposed Model

**Figure 1:**
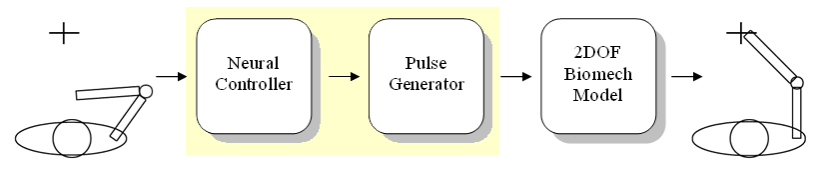
**Diagram of the modelled motor control chain**. The task is executed by the three modules, while no feedback connection is present.

Figure 1 shows a diagram of the entire model involving the cascade of the three modules.

The first module has been structured as a Multi Layer Perceptron with an architecture composed by 4 layers. The design process of the neural network used for this study is based on the analysis of the behaviour of various neural structures in responding to a same training and testing set.

In order to choose the most adequate structure, different types of neural networks have been considered and trained: a first group with only one hidden layer (varying the number of neurons), and a second group with two hidden layers (varying the number of neurons in different combinations for each layer). Experimental results considering the errors with respect to the training set and to the testing set as cross-validation (in order to avoid over-fitting problems) led us to choose an ANN design with two hidden layer of 20 neurons each.

The input layer is therefore defined by 4 input units, which correspond to the coordinates of the starting and final positions of the movement.

**Figure 2:**
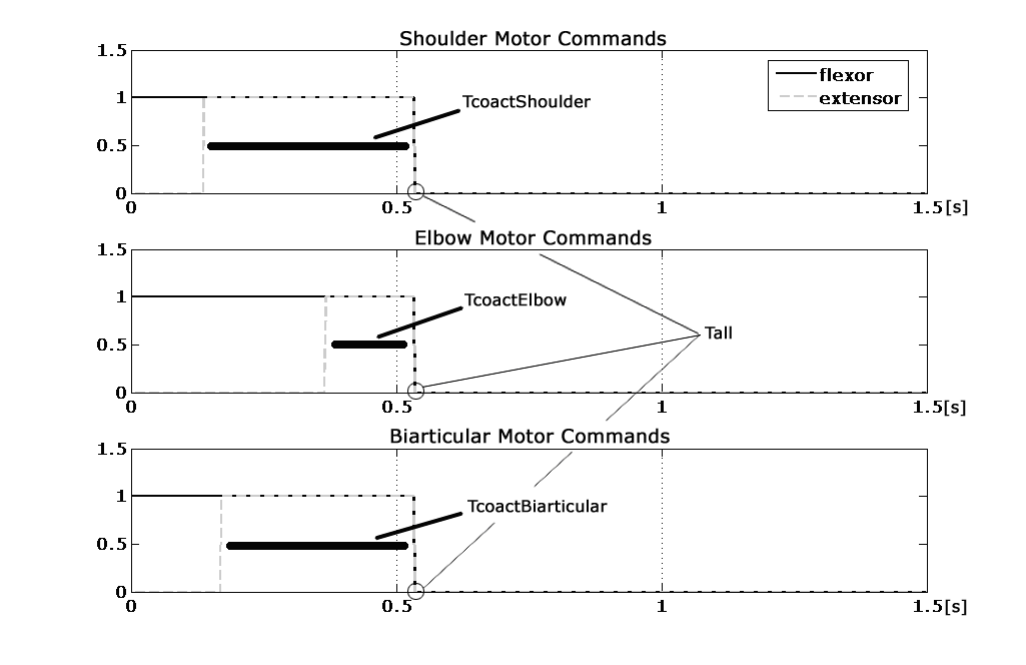
**Neural activations of the shoulder, the elbow and the biarticular muscle pair**. T_all_, total time of neural activations, is the same for all the muscles; the three T_coact _represent the interval of co-activation of flexor and extensor muscle. The value of 1.5 s in the abscissa is the total observation time.

More specifically, the first 2 units are related to the information on the initial position of the trajectory, while the other 2 units are related to the desired final position. The output layer has 4 units, because the neural network generates one value of timing for each of the three muscular pairs related to shoulder and elbow, plus one value shared by all the muscular pairs, as in fig. 2: TcoactShoulder, TcoactElbow, TcoactBiarticular, Tall, respectively. More specifically:

• for the shoulder, when the agonist muscle is activated, the movement starts. After a time interval, defined by the ANN, the antagonist is activated, so that the time interval TcoactShoulder is characterized by the co-activations of the agonist and antagonist (mono-articular) muscles of the shoulder joint (i.e. simultaneous presence of the neural inputs for shoulder muscles); its sign defines which muscle (i.e. agonist or antagonist) is activated first;

• for the elbow, TcoactElbow has the same function of TcoactShoulder;

• for the muscle pair that connect the two joints, TcoactBiarticular has the same function of TcoactShoulder and TcoactElbow.

• the movement duration is Tall: it represents the total duration of the neural activation, thus affecting the whole movement of the arm. This output value is constrained in the range 300 ms – 1 s. The time range has been chosen in order to let the limb model reach every sector of the working plane, while maintaining the ballistic characteristics of the movement.

Figure 2 depicts the profile of these neural activations having rectangular shapes, and shows the duration of the entire voluntary task ranging in the interval 300 ms and 1 s.

The transfer function chosen for every unit is the well known hyperbolic tangent nim=21+e−∑j=0Nmwjm−1⋅njm−1−1
 MathType@MTEF@5@5@+=feaafiart1ev1aaatCvAUfKttLearuWrP9MDH5MBPbIqV92AaeXatLxBI9gBaebbnrfifHhDYfgasaacH8akY=wiFfYdH8Gipec8Eeeu0xXdbba9frFj0=OqFfea0dXdd9vqai=hGuQ8kuc9pgc9s8qqaq=dirpe0xb9q8qiLsFr0=vr0=vr0dc8meaabaqaciaacaGaaeqabaqabeGadaaakeaacqWGUbGBdaqhaaWcbaGaemyAaKgabaGaemyBa0gaaOGaeyypa0ZaaSaaaeaacqaIYaGmaeaacqaIXaqmcqGHRaWkcqWGLbqzdaahaaWcbeqaaiabgkHiTmaaqahabaGaem4DaC3aa0baaWqaaiabdQgaQbqaaiabd2gaTjabgkHiTiabigdaXaaaaeaacqWGQbGAcqGH9aqpcqaIWaamaeaacqWGobGtdaWgaaqaaiabd2gaTbqabaaaoiabggHiLdWccqGHflY1cqWGUbGBdaqhaaadbaGaemOAaOgabaGaemyBa0MaeyOeI0IaeGymaedaaaaaaaGccqGHsislcqaIXaqmaaa@502D@, where the output n_i_^m ^of the i_th _neuron at the m_th _layer is obtained from the weighted outputs of the (m - 1)th level.

The values generated by the output layer, from now on indicated as neural outputs **p**, are bounded between -1 and 1, and are used by the Pulse Generator.

The system, in the present version, allows having only biphasic activation patterns for each muscle pair. Thus, the interval delimited by the initial point of the pattern and the TcoactShoulder, the TcoactElbow and the TcoactBiarticular values correspond to the Action Pulse, i.e. the time in which the neural activations of the agonist muscle determine an activation in the EMG signal, while the one going from this value till the end of the pattern, i.e. the time in which the neural co-activations of the antagonist muscle determine a braking burst in the EMG signal [21], corresponds to the Braking Command. The range of these intervals, including the co-activation time of the shoulder and the elbow muscles, together with the whole duration of the activations, establishes the direction, length and curvature of the movements.

**Figure 3:**
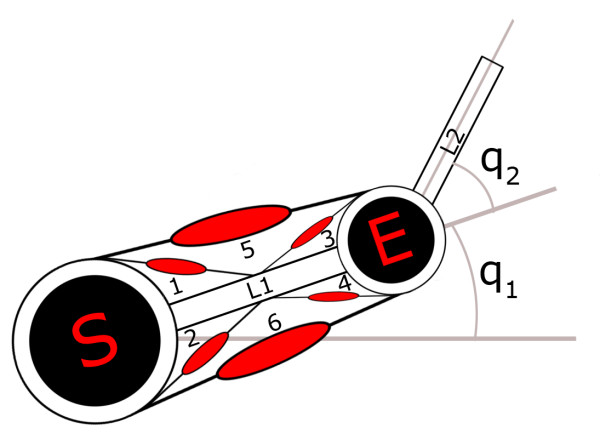
**Biomechanical model of the upper limb**. The two segments L_1 _and L_2 _represent the arm and the forearm. From the angular values q_1 _and q_2 _it is possible, by means of direct kinematics, to obtain the Cartesian position of the wrist within the working plane. The effect of gravity force is not considered in the model.

The neural outputs **p **need to be transformed in order to be utilized as commands for the muscles like mechanical actuators. Here the second module (i.e. the Pulse Generator) comes into play: its main purpose is to generate the pulse train shape, by analyzing and elaborating **p**. This pulse train should simulate the efferent commands given to the motor neurons, and thus to the biomechanical model of the arm. The third module in fig. 1 corresponds to a biomechanical model of an upper limb, composed of a skeletal structure together with a muscular structure. The skeletal model has a plant structure composed of two segments (because the wrist joint is not considered), with lengths l_1 _and l_2_, which represent the forearm and the upper arm respectively, connected through two rotoidal joints (figure 3). The planar joints that connect the two segments can assume values (q_1 _and q_2_) in the angular range [0, *π*]. These values can be put in correspondence with the Cartesian coordinates of the free end in the working plane by means of direct kinematic transformation (equation 2).

x=l1⋅cos⁡(q1)+l2⋅cos⁡(q1+q2)y=l1⋅sin⁡(q1)+l2⋅sin⁡(q1+q2)
 MathType@MTEF@5@5@+=feaafiart1ev1aaatCvAUfKttLearuWrP9MDH5MBPbIqV92AaeXatLxBI9gBaebbnrfifHhDYfgasaacH8akY=wiFfYdH8Gipec8Eeeu0xXdbba9frFj0=OqFfea0dXdd9vqai=hGuQ8kuc9pgc9s8qqaq=dirpe0xb9q8qiLsFr0=vr0=vr0dc8meaabaqaciaacaGaaeqabaqabeGadaaakqaabeqaaiabdIha4jabg2da9iabdYgaSnaaBaaaleaacqaIXaqmaeqaaOGaeyyXICTagi4yamMaei4Ba8Maei4CamNaeiikaGIaemyCaeNaeGymaeJaeiykaKIaey4kaSIaemiBaW2aaSbaaSqaaiabikdaYaqabaGccqGHflY1cyGGJbWycqGGVbWBcqGGZbWCcqGGOaakcqWGXbqCcqaIXaqmcqGHRaWkcqWGXbqCcqaIYaGmcqGGPaqkaeaacqWG5bqEcqGH9aqpcqWGSbaBdaWgaaWcbaGaeGymaedabeaakiabgwSixlGbcohaZjabcMgaPjabc6gaUjabcIcaOiabdghaXjabigdaXiabcMcaPiabgUcaRiabdYgaSnaaBaaaleaacqaIYaGmaeqaaOGaeyyXICTagi4CamNaeiyAaKMaeiOBa4MaeiikaGIaemyCaeNaeGymaeJaey4kaSIaemyCaeNaeGOmaiJaeiykaKcaaaa@6E15@

The muscular system is thus based on 6 muscle-like actuators, and establishes the dynamic relationship between the position of the arm and the torques acting on each single joint.

Body segment anthropometrics and inertias of both upper arm and forearm are obtained from the scientific literature [22], taking into account the specific body height and weight. Table 1 shows the values of the inertias adopted in the muscular-skeletal system.

**Table 1:** Numerical values of the parameters of the arm

Parameter	Units
M – Mass of the subject	80 kg
M1 – mass of the upper arm	2.24 kg
M2 – mass of the lower arm	1.92 kg
L – height of he subject	1.70 m
l1 – length of the upper arm	0.297 cm
l2 – length of the lower arm	0.272 m
I1 – inertias of the upper arm	M1*(0.322*L1)^2^
I2 – inertias of the lower arm	M2*(0.468*L2)^2^

**Figure 4:**
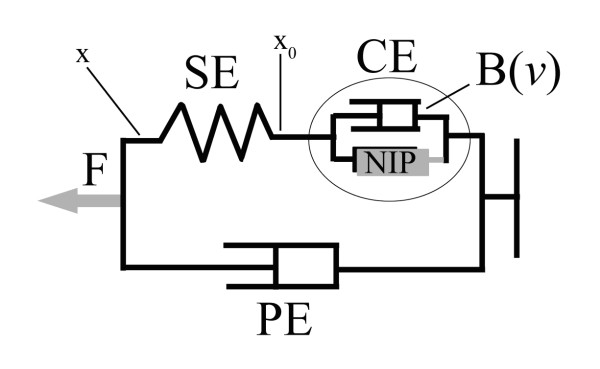
**Hill's muscle model**. The force F applied on the joint depends on SE, the series elastic element, PE, the parallel viscous element, and CE, that is the contractile element, defined by the neural input processor (NIP) and a viscous element B(ν) where ν is the shortening velocity of the muscle.

Following the work of Massone and Myers [1], each muscle is synthesized with the non-linear Hill-type lump circuit [23] as depicted in figure 4.

According to the notation present in [9], the neural outputs serve as inputs for the actuator, resulting in a time function called F_0 _representing the muscle tension. The Hill model is composed of a series elastic element (SE), a parallel viscous element (PE) and a contractile element (CE) which includes the non-linear viscosity B depending on the shortening velocity ν, as in equation 3

B={(a⋅T0)/(b+v)a'⋅T0v≤0v>0a=a'=4,b=1
 MathType@MTEF@5@5@+=feaafiart1ev1aaatCvAUfKttLearuWrP9MDH5MBPbIqV92AaeXatLxBI9gBaebbnrfifHhDYfgasaacH8akY=wiFfYdH8Gipec8Eeeu0xXdbba9frFj0=OqFfea0dXdd9vqai=hGuQ8kuc9pgc9s8qqaq=dirpe0xb9q8qiLsFr0=vr0=vr0dc8meaabaqaciaacaGaaeqabaqabeGadaaakeaacqWGcbGqcqGH9aqpdaGabeqaauaabeqabmaaaeaafaqabeGabaaabaGaeiikaGIaemyyaeMaeyyXICTaemivaq1aaSbaaSqaaiabicdaWaqabaGccqGGPaqkcqGGVaWlcqGGOaakcqWGIbGycqGHRaWkcqWG2bGDcqGGPaqkaeaacqWGHbqycqGGNaWjcqGHflY1cqWGubavdaWgaaWcbaGaeGimaadabeaaaaaakeaafaqabeGabaaabaGaemODayNaeyizImQaeGimaadabaGaemODayNaeyOpa4JaeGimaadaaaqaaiabdggaHjabg2da9iabdggaHjabcEcaNiabg2da9iabisda0iabcYcaSiabdkgaIjabg2da9iabigdaXaaaaiaawUhaaaaa@56AF@

where a, b and a' are constant parameters (whose measurement units are respectively a = [m^-1^], b = [rad/s] and a' = [a/b]) and T_0 _is the value of the torque applied by the single muscular unit as a percentage of the maximum isometric force associated to that muscle (T_0 _= Fmax*F_0 _*d, where d is the average moment arm, Fmax is the maximum isometric force associated to that muscle and F_0 _is the percentage coefficient), thus resulting in a different behaviour of the contractile element when shortening or lengthening. Table 2 shows the numerical values of the parameters of the Hill's model.

**Table 2:** Numerical values of the Hill's parameters

**Parameter**	**Units**
Kse	120 N/rad
Bpe	30 N.s/rad
Fmax(shoulder)	800 N
Fmax(elbow)	700 N
Fmax(double joint)	1000 N

The force difference between the muscles of each single joint is implemented on the actuators by means of different maximal amplitudes of the corresponding forces. The values of the forces are related to maximal values that are represented in Table 2. Then the effects of the corresponding torques thus obtained are then summed in order to obtain the overall torques on each joint *τ*_1 _and *τ*_2_, as in Equation 4:

τ1=F1−flex−F1−ext+φ⋅F3−flex−φ⋅F3−extτ2=F2−flex−F2−ext+ϕ⋅F3−flex−ϕ⋅F3−ext
 MathType@MTEF@5@5@+=feaafiart1ev1aaatCvAUfKttLearuWrP9MDH5MBPbIqV92AaeXatLxBI9gBaebbnrfifHhDYfgasaacH8akY=wiFfYdH8Gipec8Eeeu0xXdbba9frFj0=OqFfea0dXdd9vqai=hGuQ8kuc9pgc9s8qqaq=dirpe0xb9q8qiLsFr0=vr0=vr0dc8meaabaqaciaacaGaaeqabaqabeGadaaakeaafaqabeGabaaabaacciGae8hXdq3aaSbaaSqaaiabigdaXaqabaGccqGH9aqpcqWGgbGrdaWgaaWcbaGaeGymaeJaeyOeI0IaemOzayMaemiBaWMaemyzauMaemiEaGhabeaakiabgkHiTiabdAeagnaaBaaaleaacqaIXaqmcqGHsislcqWGLbqzcqWG4baEcqWG0baDaeqaaOGaey4kaSIae8NXdyMaeyyXICTaemOray0aaSbaaSqaaiabiodaZiabgkHiTiabdAgaMjabdYgaSjabdwgaLjabdIha4bqabaGccqGHsislcqWFgpGzcqGHflY1cqWGgbGrdaWgaaWcbaGaeG4mamJaeyOeI0IaemyzauMaemiEaGNaemiDaqhabeaaaOqaaiab=r8a0naaBaaaleaacqaIYaGmaeqaaOGaeyypa0JaemOray0aaSbaaSqaaiabikdaYiabgkHiTiabdAgaMjabdYgaSjabdwgaLjabdIha4bqabaGccqGHsislcqWGgbGrdaWgaaWcbaGaeGOmaiJaeyOeI0IaemyzauMaemiEaGNaemiDaqhabeaakiabgUcaRiab=v9aQjabgwSixlabdAeagnaaBaaaleaacqaIZaWmcqGHsislcqWGMbGzcqWGSbaBcqWGLbqzcqWG4baEaeqaaOGaeyOeI0Iae8x1dOMaeyyXICTaemOray0aaSbaaSqaaiabiodaZiabgkHiTiabdwgaLjabdIha4jabdsha0bqabaaaaaaa@8A7B@

where Φ = 0.6 and *ϕ *= 0.4 are non dimensional units and the F values in the equation are the values of the torque applied by each muscle of the corresponding joint during flexion or extension.

Finally, the trajectory in the working plane is obtained from a double integration at each sampling time of the acceleration of the end point of the effector due to the changes in the overall torque applied to both joints.

### The Learning Paradigm

One key point of the present work is the training paradigm adopted for the neural controller with the aim of defining a specific internal model during ballistic movements of the arm, that is to establish a mapping between the desired movements within the working plane and the necessary neural outputs, so that the controller could learn the inverse dynamics of the biomechanical arm model. The algorithm will adapt the neural weights and biases so that, if the 4 inputs of the network respectively correspond to the coordinates of the starting point [q_1_, q_2_], and of the desired target [q_1_^d^, q_2_^d^], then the output of the net will approach the correct **p**.

**Figure 5:**
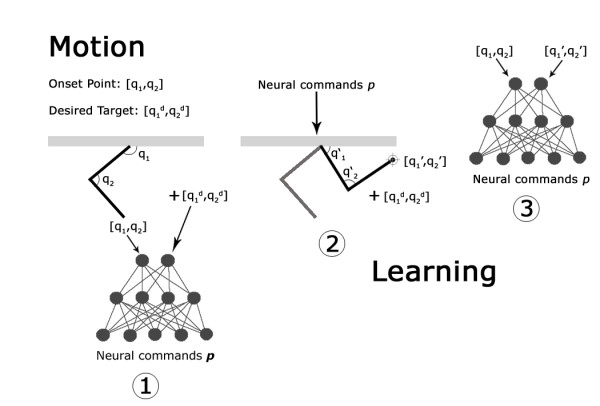
**Diagram of the exploration and the learning process**. (1) The arm starts in the position defined by the angle q_1 _and q_2_(Cartesian position x_s_, y_s_), while the desired target position is defined by q_1_^d ^and q_2_^d ^(Cartesian position x_d _and y_d_). The angles q_1_' and q_2_' univocally define the spatial configuration of the arm in the arrival point (Cartesian position x_a_, y_a_) (2), that in the early phases of the learning process is different from the desired one: the ANN learns the association between the starting point and the arrival point (3).

More precisely, as shown in the scheme depicted in figure 5, the output **p **of a non-trained network (phase 1) can be the input for the biomechanical arm model (phase 2): this input leads to the execution of a reaching movement in general different from the desired one, that is towards a different target. These neural inputs **p**, together with the starting and ending points coordinates, become the new data for the training of the network (phase 3). In this way, a mapping between muscular activations and points of the working space can be attained.

The key feature of this approach is that the position error in executing the movements is not used in the training. The reason is that, following the studies of [20] a supervised training mechanism for the controller must be excluded, thus meaning that the knowledge of the position error made in carrying out the movement will not be used to train the neural network. The exclusion of a feedback circuit both in the phases of learning and executing the task, reflects the capacity of the motor control system to explore the workspace either without basing itself on pre-existent information (batch supervised training) or elaborating the data coming from the environment (feedback error learning). In the learning phase of the network, the association: "starting point – neural inputs generating the movement from the starting point to an ending point" is therefore used. This is the step-by-step procedure in which the controller learns to make different movements.

It is important to stress again that, unlike most of the models proposed in the literature, this controller learns the movement actually carried out, not the wanted one. This training strategy recalls the big picture of the classical Piagetian's concept of motor development. More in particular it can be considered as leading the way to the circular reaction learning model. Otherwise, in the proposed scheme, the construction of the inverse dynamics of the arm within a particular environment neglects the interconnection between the eye and the arm systems, but is driven by a purely proprioceptive exploration phase outlining the development of an internal model. During the training phase, the neural controller tends to achieve an optimal behaviour in reaching a desired target point by improving the correlation between the sensory map (starting and ending point) and the motor map (muscular activations which generate the movement between these two points) through the entire working plane. The reduction of the error on the final position can be thus considered as a consequence and not a cause of the learning procedure. The proposed neural model, basing on the philosophy architecture of Direct-Inverse Model (Jordan, 1995), shows novel and innovative characteristics.

### Simulating the Internal Model: the training phase

During the training, the system automatically and randomly chooses the starting and ending points of the movements, which in turn determine the parameters **p **to be used in the Pulse Generator.

In addition, during the training a random noise generator acts on the output of the neural network in order to prevent convergence on local minima, which would imply a limitation in direction or amplitude of upper limb movements.

**Figure 6:**
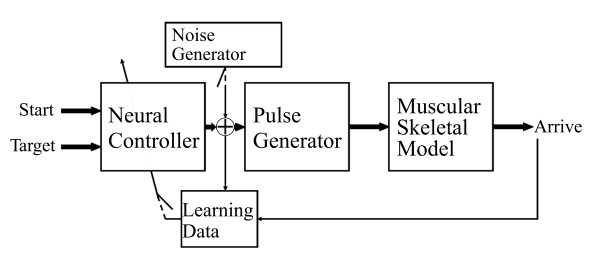
**Learning scheme of the proposed model**. The noise is added to the neural input generated by the controller. The new vector *n*_*i *_is thus used for the generation of the muscular activities and for the controller training process.

In fact, especially for the very first period of exploration, is it possible to have small variations in the weights of the neural controller. This could possibly bring the neural network to converge to a local minimum state, where the weights are not optimally calibrated to face the problem of the arm control. For this reason, the noise generator intervenes on the output parameters **p **of the neural controller with a probability exponentially decreasing with the number of overall movements (see figure 6).

In the initial phases of the training, the controller is not trained, and there is no correspondence between the desired target and the one actually reached by the movement of the biomechanical model of the arm. At the end of each task, a standard back-propagation algorithm with momentum is used for the training and thus the variation of the weights.

The training of the artificial neural network and the complete coverage of the working plane, with respect to both the possible starting and target points, can be reached with about 200.000 random generations (epochs). The decision about the end of the training is not based on a prefixed number of movements/training steps but on the monitoring of the convergence of the network.

Once the neural controlled is trained, the overall system is tested and the behaviour is analyzed. In this second phase, the noise generator is not active. Even if the inputs driving the network are different from those used in the training phase, the generalization capabilities of the connectionist system enables it to operate correctly.

### Simulating the Internal Model: Testing the performance of the model

Tests, and comparisons with results available in literature have been performed in order to evaluate the performance of the model after the convergence of the network.

The neural controller has been tested by presenting a high number of pairs of randomly chosen start-target points, and the errors in reaching the target have been recorded. Initially, in order to qualitatively test the behaviour of the controller, a set of 1000 movements starting from the same initial point have been considered. This set have been used to analyze the capacity to cover the entire workspace, to give a graphical representation of the correlation of the error position with respect to the length of the movements and to observe the distribution of the peak velocity within the working plane.

Furthemore,1200 random movements ranging from 5 cm to 60 cm, subdivided into groups spaced out by 5 cm (200 movements per group), have been generated, in order to make a comparison with the kinematic analysis of ballistic arm movements presented in literature (such as in [8,14,24]), where movements with a maximum amplitude of ± 30 cm have been examined. This subset has been defined Physiological Subset (PS). The characteristics of these tasks have been analyzed and compared to the data obtained from experimental tests on human beings, carried out in [8,24]. In the latter paper, indexes useful to quantitatively determine some characteristics of the movements have been calculated.

The accuracy of the neural network in implementing the movements has been characterised by means of the following parameters:

• The absolute position error of the arrival position reached by the end-effector with respect to the desired final position (or target).

• The module error (the amplitude error).

• The phase error (the error pointing at the target).

• The curvature.

• The velocity curve.

The position and phase errors have been chosen in order to reveal the presence of a biased behaviour. In particular, the module error |e| has been defined as the difference between the segment connecting the starting point and the arrival point (x_a_, y_a_) and the straight line from the starting point to the target (x_t_, y_t_).

**Figure 7:**
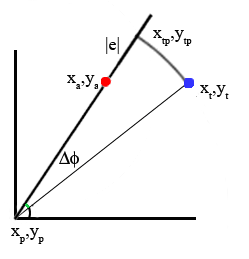
**Module and Phase Error**. Considering the movement directed from the starting point (x_p_, y_p_) to the arrival point (x_a_, y_a_), the module error (or amplitude error) |e| is the distance between (x_tp_, y_tp_) and (x_a_, y_a_); the phase error (or the direction error) is the angular difference between the segment connecting (x_p_, y_p_) and (x_t_, y_t_) and the segment connecting (x_p_, y_p_) and (x_a_, y_a_).

The phase error ∠e (Δ*ϕ*) has been defined as the difference of the angles which identify the two lines connecting the starting point with respectively the target and the arrival point, and it has been used to determine if the neural controller was able to correctly point at the target. The pair of error parameters are graphically explained in figure 7.

**Figure 8:**
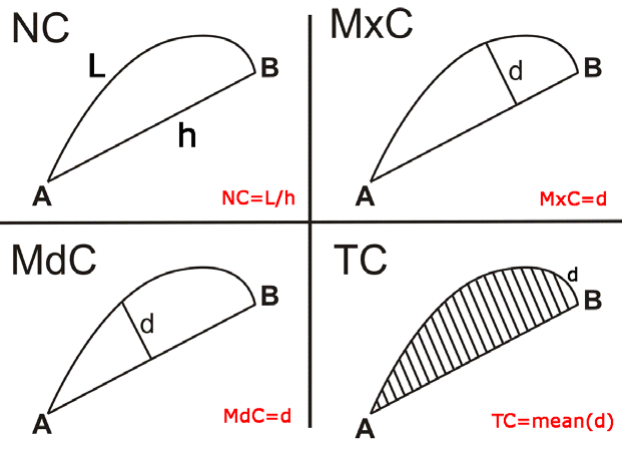
**Curvature Indexes**. The figure shows the 4 indexes taken into account: the Normal Curvature (NC) is the ratio between the length of the trajectory executed (L) and the straight line connecting the starting point and the arrival point (h). The Maximum Curvature is the maximum distance (d) between L and h. The Medium Curvature is the distance (d) between L and h evaluated in h/2. In the end the Total Curvature is the mean value of all the distances d between L and h.

For the curvature, there are various definitions in the literature. The index of curvature of a movement, C, is defined in [24] as the ratio between the curvilinear abscissa and the minimum Euclidean distance between the starting and the arrival point.



where the numerator represents the amplitude of the movement carried out, while the denominator is the minimum distance between the starting point and the arrival point. This is defined as the Normal Curvature (NC). In [25,26], two curvature indexes are used: the first is the ratio between the distance from the medium point of the straight line connecting the starting (A) and the arrival point (B) and the trajectory performed by the subject (medium curvature: *MdC*), while the second considers the maximum value of all the distances from the points defining the trajectory and the straight line defining the minimum distance from the two extremities of the path (maximum curvature: *MxC*). In [27] the measure of curvature is obtained from *MxC*, by replacing the maximum value with the mean value (total curvature: *TC*). Figure 8 graphically describes these differences.

The coefficient of variation (CV), defined as the ratio between the standard deviation and the mean error position has also been evaluated. The distribution of the neural activation times with respect to the length of the movements has been taken into account.

Finally, the performance of the model with respect its possibilities of adapting to modifications in the environment, such as the presence of disturbing force fields, has been taken into account. To this purpose, a force proportional to the movement speed and directed along the horizontal axis has been inserted in the model, after the training for unobstructed movements in all the working plane. The additional training necessary to the model to be able to cope with this force and the performance as for the reaching errors have been evaluated.

## Results and Discussion

The proposed neural system is able to achieve a complete coverage of the working plane, unlike other models [9] which are limited to short amplitude motor tasks, usually around 20–30 cm.

**Figure 9:**
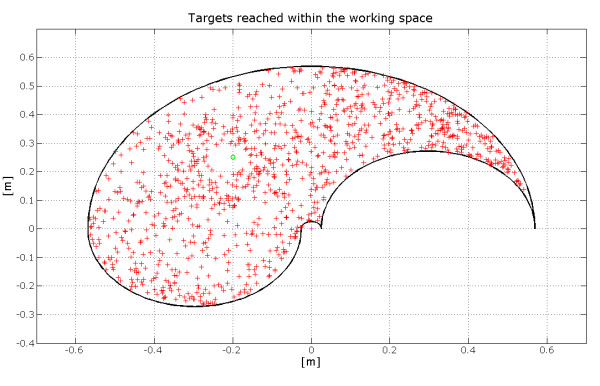
**Distribution of the targets reached within the working plane**. The starting point is indicated with the circle mark. It is possible to observe an almost complete coverage of the area.

This feature can be appreciated in figure 9 where, for visualization purposes, the same starting point and 1000 target points have been considered.

**Figure 10:**
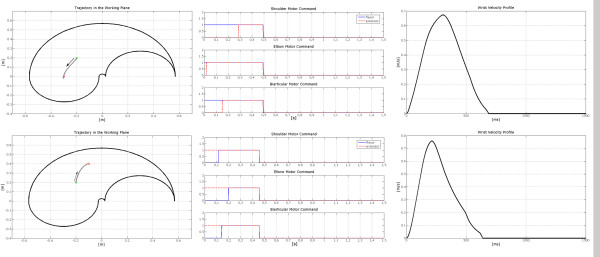
**Example of two movements carried out by the arm model guided by the trained neural controller starting from the same initial position**. The starting point is the same for the 2 tasks (coordinates: x = -0.2; y = 0.2); the arrival points have been chosen in 2 different symmetric positions with respect to the starting point, at a distance of about 22.4 cm. Each row represents a different movement. The left column of this image depicts the trajectory followed by the wrist. The central column shows the neural inputs necessary for the motor commands of the flexor and extensor muscles acting on both the shoulder and the elbow joint. The right column shows the wrist velocity profile.

Figure 10 shows two different movements starting from the same point, together with the neural outputs **p **and the relevant velocity profiles.

The first movement of the set simulates the role of the Pectoralis Major, in the shoulder joint, for targets positioned in a position west with respect to the starting point, while the second one implies the use of the Deltoid for the target allocated in a position east with respect to the starting point. The velocity profile reflects the bell shaped behaviour typically found in literature (see e.g. [14]).

**Figure 11:**
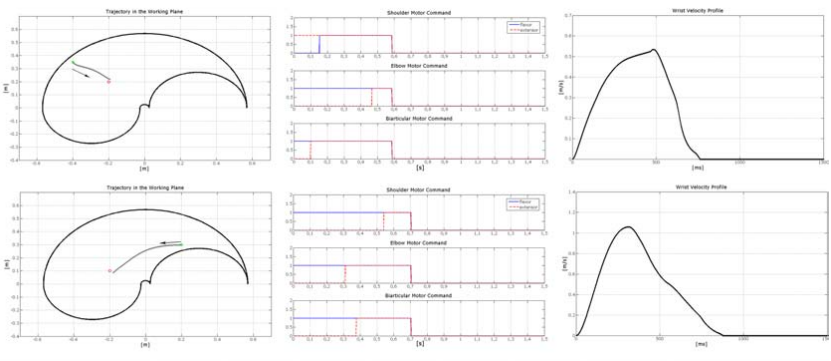
**Example of two movements carried out by the arm model guided by the trained neural controller starting from different initial positions**. The two tasks start from different points, and point towards different directions within the working plane. In the upper row, the central column shows the neural commands of the muscle pair of the shoulder and of the elbow joint necessary for the trajectory presented in the left column. The movement starts at the point [-0.4; 0.35] while the target point is at [-0.2; 0.2]. In the lower row, the right column shows the wrist velocity profile for the second movement whose starting point is at [-0.2; 0.3] and whose target point is at [-0.2; 0.1].

Figure 11 shows that even when changing the starting point, the relations between the direction of the movement and the neural inputs persist.

**Figure 12:**
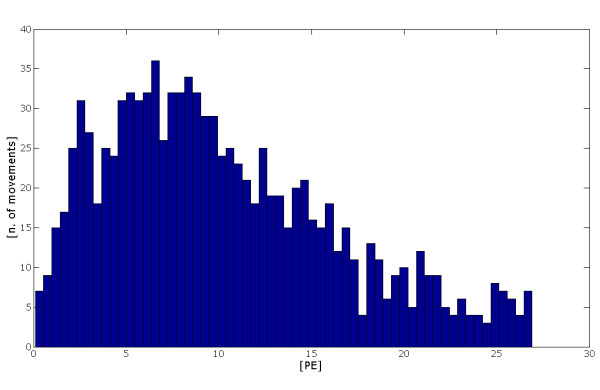
**Histogram of the percentage error position**. Histogram of the percentage error position (PE, that is the absolute position error with respect to the length of the movement carried out) of the end effector of the upper limb with respect to the number of movements analyzed.

For the PS, the mean position error has been of about 4.8 cm with a standard deviation of about 4 cm. Figure 12 shows the histogram of the percentage of the absolute position error with respect to the length of the movement. The mean absolute error, normalised with respect to the length of the movements, resulted always lower than 0.27. These findings show that the model is able to accurately simulated ballistic (unobstructed) movements of the arm.

**Figure 13:**
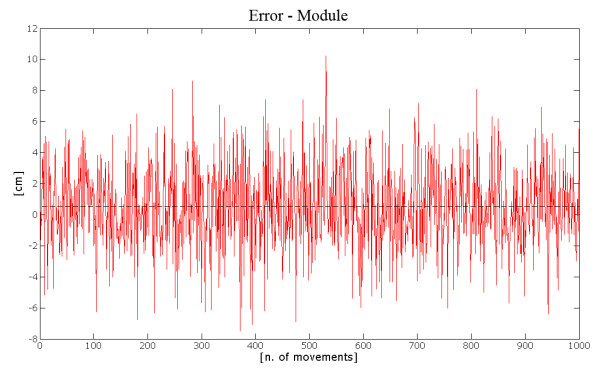
**Module Error**. The figure shows the trend of the module error (or amplitude error) with respect to the movements included in the subset analyzed. It is possible to observe that the mean value is close to zero, thus proving an unbiased behaviour.

**Figure 14:**
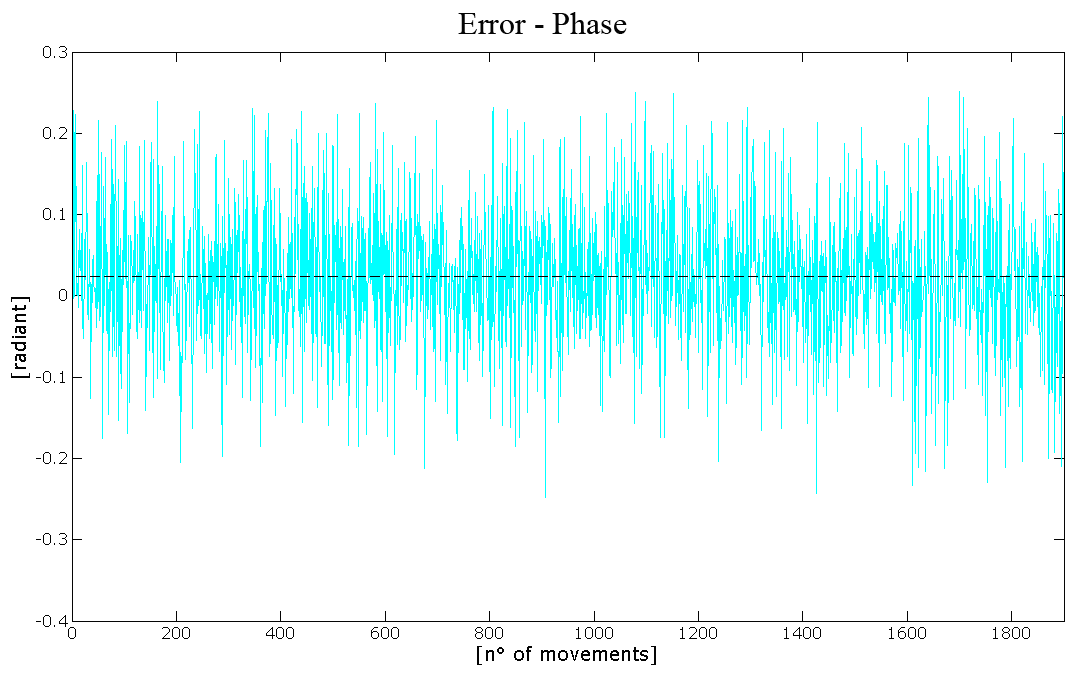
**Phase Error**. The figure shows the trend of the phase error (or direction error) with respect to the movements included in the subset analyzed. Also in this case the mean value is negligible.

The module error shows a value of 0.51 cm., as illustrated by Figure 13. The mean value of the angular error, presented in figure 14, resulted almost negligible, thus showing that the ANN gives unbiased results, that is it is able to correctly point (in the average) at the target with limited (in the average) errors.

**Figure 15:**
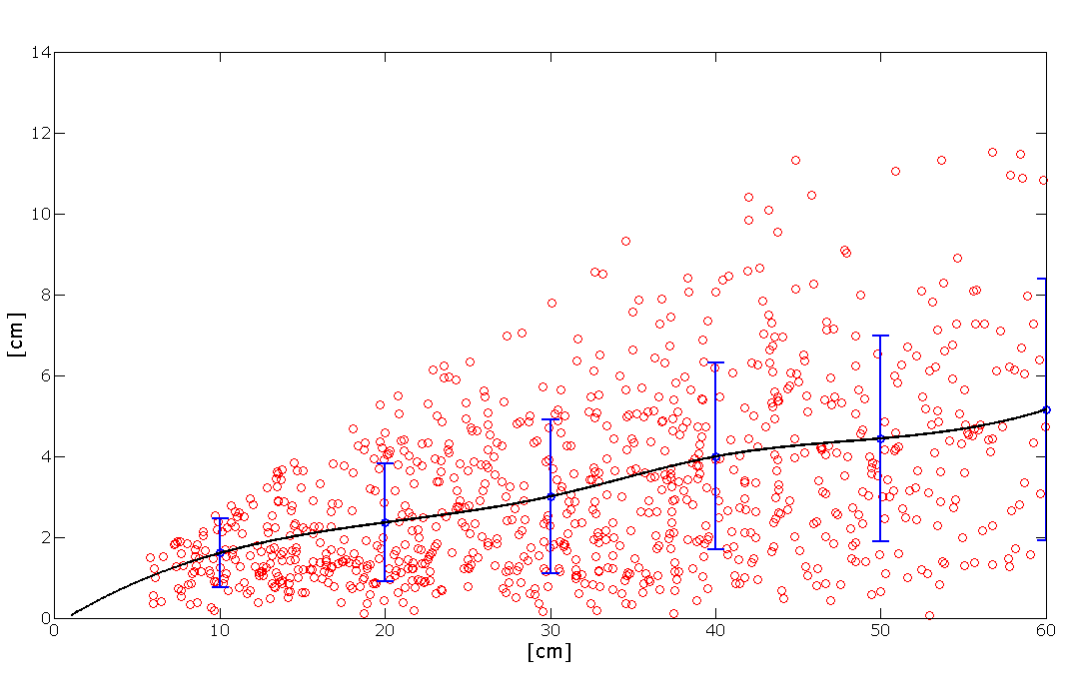
**Dispersion of the error with respect to the length of the movements**. It is possible to observe a monotonic increase of the mean position error value with respect to the length of the movement. The vertical bars represent the value 2*STD.

Moreover, in figure 15 it is possible to see that the mean absolute position error has a limited variation with the increase of the movement length.

When analysing the CV of the movements in PS it is possible to observe that monotonically increases ranging from 0.6 to 0.8. This behaviour can be explained by considering that when the movement becomes longer the precision in reaching the target decreases and the position error distribution increases. A comparison between the experimental data reported in [24,26] and the data extracted from the simulated model of the present work is interesting because it puts in evidence the behaviour of the proposed neural model for as what concerns the curvature.

To compare our results with the data in the literature, the four values of curvature have been taken into account. The table 3 shows the mean values of *NC*, *MxC*, *MdC *and *TC*). The mean value of *NC *reported in [28] is about 1.02, for movements with a maximum amplitude of 42 cm, while in this system the mean value is 1.06.

**Table 3:** Mean values of the curvature indexes for the set of movements

Normal Curvilinearity NC	1.09
Maximum Curvilinearity MxC	0.63 cm
Medium Curvilinearity MdC	0.61 cm
Total Curvilinearity TC	0.16 cm

Two main things must be stressed out:

• even if the biomechanical arm model is only an approximation of a real upper limb structure, in which further muscle activations have an influence, even if minor, on the overall movement, the results are very interesting.

• all the experiments on human subjects from the literature are replications of the same set of movements in different direction or with different amplitude; this brings a specialization of the tasks during the trials and therefore to lower errors.

In [26,29] the normalized maximum curvature shows a value of about 0.05 ± 0.02. This result has been estimated as the ratio between the maximum distance from the straight line connecting the starting and the arrival point (that is the value *MxC *of the present system) and the length of straight line connecting them; moreover the values reported are related to tasks performed on the sagittal plane.

**Figure 16:**
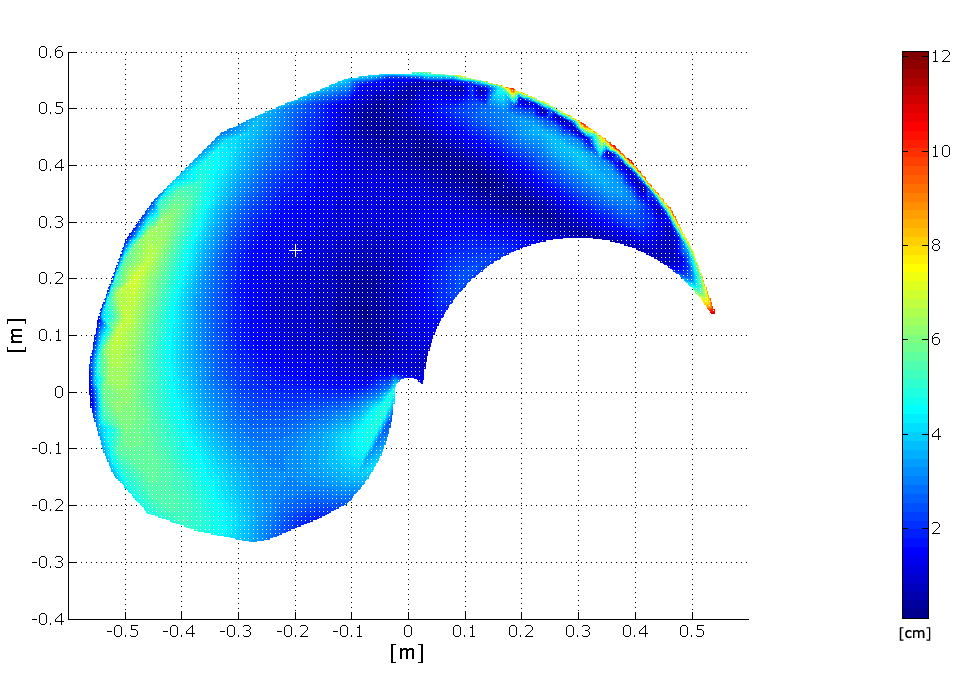
**Distribution of the absolute error position within the working plane**. The figure shows that higher values are mostly present along the borderline of the working plane.

Figure 16 depicts a bi-dimensional projection of the error for the wrist final position when implementing 1000 test movements, with the same starting point. Taking off the outliers (which are the movements that show a ratio between final error position and length of the desired task greater than 27%), the results considering only one starting point and movements with a maximum amplitude of 60 cm show a mean error position value of about 2.4 cm with a standard deviation of 1.8 cm (it is possible to see that the behaviour is quite uniform, even if there are some error peaks far from the starting point that can justify a correlation different from zero).

**Figure 17:**
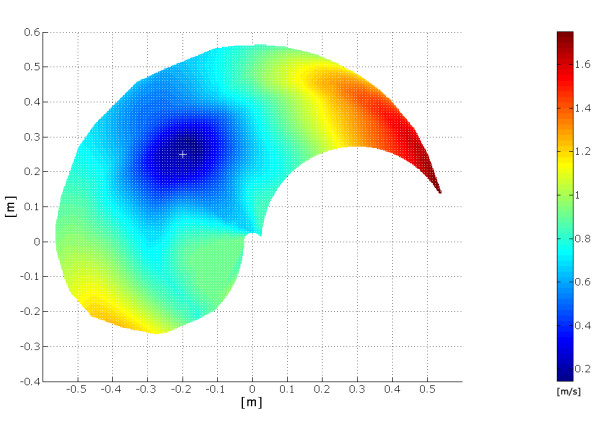
**Graph of the scale effect**. The figure shows the distribution of the wrist peak velocity with respect to the distance from the starting point. It is possible to observe a uniform increase of the peak velocity from the area near the starting point to the borders of the working plane.

Figure 17 shows the behaviour of the velocity profile whose peak value, when considering the movements starting from the same point, increases accordingly with the length of the movements. From the model it has been possible to evaluate the presence of the "scaling effect" which explains the invariant property of the wrist velocity profile: when the length of the movement increases, so does the maximum velocity reached along the trajectory while maintaining the same profile.

**Figure 18:**
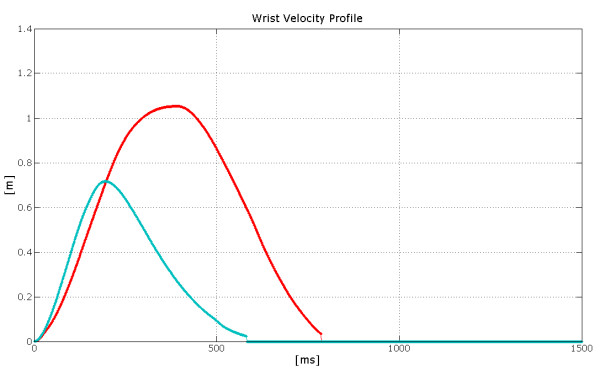
**Comparison between wrist velocity profile**. The figure shows the wrist velocity profiles of two different movements starting from the same initial point, directed towards the same direction but with different amplitudes. Shorter movement is related to the slower velocity profile (the blue one).

Figure 18 shows that the velocity curve maintains the same profile for shorter and larger movements, and that the duration of the movements does not increase linearly with their length. These findings are similar to those present in [14,23].

**Figure 19:**
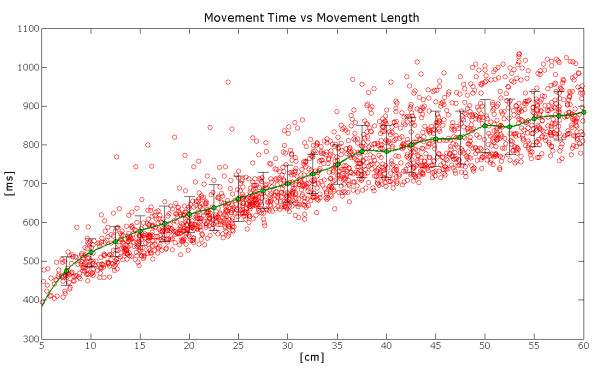
**Dispersion of the neural activation times with respect to the length of the movements**. From the figure it is possible to observe an increment of he neural activation time.

Moreover, similar activations bursts are associated to similar movements: i.e. it is possible to see that in movements directed towards the same area inside the working plane, not only the same muscles of the shoulder and the elbow joint are activated first, but also the intervals of the neural activations of these muscles show the same duration. This finding can be correlated with a feature that could be defined as a global isochrony of the movements. In Figure 19 the value of the total activation time of activation with respect to length of the movement is shown. It emerges that the time spent increases, though not proportionally, to the length of the movement.

**Figure 20:**
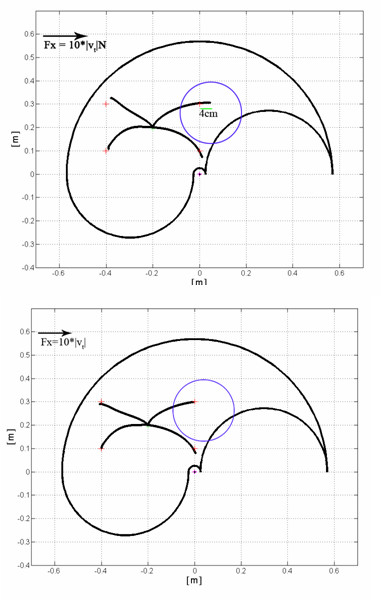
**Adaptation of the neural controller to external forces**. The upper figure shows the effect of a force, directed along the x-axis, applied on the end-point of the biomechanical arm model and proportional to its velocity. The shift from the arrival point induced by the disturbances is clearly visible. The lower figure shows the behaviour of the overall system after a training period of the neural controller within he modified working space. The controller learns how to take into account the effects of the external forces acting on the dynamics of the arm model.

Finally, we simulated the insertion of the model in a force field, proportional to the movement speed, with a peak amplitude up to 15 N and directed along x axis, which acts on the already trained controller. The additional training needed by the model to be able to cope with this force required only the 1% of the epochs necessary for the training all over the working plane for unobstructed movements. It resulted that the model learned to deal with this force by modifying the activation intervals of the muscles, thus increasing the stiffness of the arm through co-contractions of the muscles. After the additional training, in the testing phase, the model showed errors similar to those obtained with no force. In figure 20 it is possible to observe the behaviour of the system in the force field and after the short re-learning phase in the new environment.

## Conclusion

A neural-network motor controller able to simulate the ballistic movements of an arm has been presented. This controller is implemented by means of a neural network that simulates the internal model devoted to the management of the feed-forward aspects of the movement. The biomechanical model includes three pairs of muscles, and two joints.

The results obtained are plausible from a biological standpoint and might be interpreted taking into account some features:

• the capability of the controller to solve the inverse dynamics problem, that is to generate the proper muscular activations and then the muscular forces, exclusively on the basis of kinematic information such as the starting and ending point of the movements;

• the capacity of the neural controller to acquire the internal model of the plant with a learning process that excludes the use of an online feedback on the position error, thus showing a biologically plausible behaviour;

• the ability of the overall system to obtain realistic trajectories and bell shaped profiles similar to the experimental ones: the value of the parameters characterising the trajectories are in good agreement with those obtained from experiments on humans in similar tasks;

• the paradigm adopted for the on-line learning of the system dynamics that includes the biomechanical characteristics of the arm. In this way, both the adaptive characteristics of the controller with respect to the plant, and the simplicity of the control activations are emphasised.

Even if the model can be further complicated by optimizing the biomechanical model, to increase its capacity to obtain realistic trajectories, the presented results open a wide field of applications: from the cognitive ones to the use of this model for the control of smart Functional Electrical Stimulation (sFES) systems, to the rehabilitation. For instance, the availability of such a controller, once adapted to electrical stimulation systems, could enhance the possibilities of paretic patients to control their arm movements with more reduced effort during rehabilitation sessions, thus stimulating cortical synaptic plasticity and the recovery of correct muscular synergies.

The availability of such a controller, once adapted to electrical stimulation systems, can greatly enhance the possibilities of paretic patients to control their arm movements with more reduced effort during rehabilitation sessions, thus stimulating cortical synaptic plasticity and the recovery of correct muscular synergies. Such an application is quite new because most paretic patients have neural implants for grasping, while better and more physiological movements imply the involvement also of the entire arm. This controller not necessarily must rely on implanted cuff electrodes and could use surface and/or minimally invasive stimulators. The system can be driven by some HCI (for instance, a gaze tracker) which gives the neural controller the intention of the movement (starting and ending points) leaving the burden of activating the stimulation to the neural net. Obviously, the system proposed is able only to make ballistic planar movements, which in any case constitute as a proof of concept.
